# Functional Interactions between 17**β**-Estradiol and Progesterone Regulate Autophagy during Acini Formation by Bovine Mammary Epithelial Cells in 3D Cultures

**DOI:** 10.1155/2014/382653

**Published:** 2014-05-07

**Authors:** Katarzyna Zielniok, Tomasz Motyl, Malgorzata Gajewska

**Affiliations:** Department of Physiological Sciences, Faculty of Veterinary Medicine, Warsaw University of Life Sciences, Nowoursynowska 159, 02-776 Warsaw, Poland

## Abstract

Mammary gland epithelium forms a network of ducts and alveolar units under control of ovarian hormones: 17-beta-estradiol (E2) and progesterone (P4). Mammary epithelial cells (MECs) cultured on reconstituted basement membrane (rBM) form three-dimensional (3D) acini composed of polarized monolayers surrounding a lumen. Using the 3D culture of BME-UV1 bovine MECs we previously demonstrated that autophagy was induced in the centrally located cells of developing spheroids, and sex steroids increased this process. In the present study we showed that E2 and P4 enhanced the expression of *ATG3*, *ATG5*, and *BECN1* genes during acini formation, and this effect was accelerated in the presence of both hormones together. The stimulatory action of E2 and P4 was also reflected by increased levels of Atg5, Atg3, and LC3-II proteins. Additionally, the activity of kinases involved in autophagy regulation, Akt, ERK, AMPK, and mTOR, was examined. E2 + P4 slightly increased the level of phosphorylated AMPK but diminished phosphorylated Akt and mTOR on day 9 of 3D culture. Thus, the synergistic actions of E2 and P4 accelerate the development of bovine mammary acini, which may be connected with stimulation of *ATGs* expression, as well as regulation of signaling pathways (PI3K/Akt/mTOR; AMPK/mTOR) involved in autophagy induction.

## 1. Introduction

Functional development of the mammary gland is a process composed of a sequence of strictly regulated events that start early in the foetal life and end in mature females at the beginning of lactation. The first quiescent phase of mammary growth is characterized by the formation of rudimentary system of ducts from ectodermal epithelium during embryogenesis and after birth. More intensive phase of mammary development takes place at the onset of puberty and is manifested by the extension and branching of ducts, which is coincident with growth of mammary fat pad, but in the absence of alveolar structures. However, the majority of morphological and physiological development of the mammary gland takes place during pregnancy, when the parenchymal tissue undergoes extensive remodelling. Replacement of adipose tissue by parenchyma and condensation of stromal connective tissue to narrow bands are accompanied by alveologenesis formation of differentiated alveoli required for milk production [1]. The process of secretory epithelium division continues into the early stages of lactation and is terminated with the epithelial tissue regression occurring at involution. All stages of mammary gland development are under strict control of endocrinal factors: reproductive and metabolic hormones, as well as growth factors secreted locally in paracrine or autocrine manner [2].

*Sex Steroids*. 17*β*-Estradiol (E2) and progesterone (P4) are essential regulators of mammary gland development and are necessary to fulfill proper lobuloalveolar structure formation. While estradiol-mediated signaling plays a major role in ductal morphogenesis, progesterone is critical for development of lobuloalveolar structures [3]. 17*β*-Estradiol and progesterone mediate their biological responses mainly by their specific receptors (two forms of estradiol receptor, ER*α* and ER*β*, and progesterone receptor PR) via genomic pathway. The expression and amount of these receptors in the mammary epithelial cells (MECs) undergo regulatory changes during mammary growth and involution [4]. The growing importance of sex steroids action in mammary gland development lies in their property of regulating crucial cellular processes such as growth, differentiation, apoptosis, and autophagy [3, 5–8].

Macroautophagy (herein referred to as autophagy) is a highly conserved cellular self-degradative pathway, activated under various stress conditions as an adaptation to nutrient deprivation, and used for energy and macromolecules generation to maintain cellular homeostasis [9]. We have previously reported that 17*β*-estradiol and progesterone positively regulate autophagy during formation of acinar structures by bovine mammary epithelial cells (MECs) cultured in three-dimensional (3D) system on reconstituted basement membrane (rBM), commercially available as Matrigel [8]. Bovine BME-UV1 mammary epithelial cells cultured on Matrigel form proper acinar structures within 16 days, and their development is enhanced in the presence of sex steroids: 17*β*-estradiol and progesterone.* In vitro* studies have demonstrated that autophagy is activated in the center of mammary acini developing on rBM. This process is preceded with polarization of cells that are in direct contact with extracellular matrix components but is induced prior to apoptosis, which is the main form of cell death responsible for lumen clearance, determining the proper development of mammary alveoli [10, 11]. Induction of autophagy in MECs is closely connected with the lack of contact with extracellular matrix and exposure to nutrient deprivation conditions, contributing to survival of cells during anoikis (cell detachment-induced apoptosis) [11]. Our research showed that in the presence of sex steroids, autophagy was augmented in the developing acinar structures. Therefore, in the present study we used 3D culture model to elucidate the mechanisms of autophagy regulation by E2 and P4 during formation of alveoli-like structures by bovine BME-UV1 mammary epithelial cells. We investigated the genomic effect of both sex steroids on the expression of chosen autophagy-related genes (*ATGs*):* ATG3, ATG5, BECN1, LC3B,* and their protein products. Additionally we examined activation of Bcl-2 and different kinases: Akt, Erk, AMPK, and mTOR, which are known to regulate autophagy, in order to determine whether the effect of E2 and P4 is also connected with the nongenomic actions of these steroid hormones.

## 2. Material and Methods

### 2.1. Media and Reagents

DME/F-12, RPMI-1640, NCTC 135, *α*-lactose, lactalbumin hydrolysate, glutathione, bovine insulin, bovine holo-transferrin, hydrocortisone, L-ascorbic acid, insulin-like growth factor-I (IGF-I), 17*β*-estradiol (E2), PI3K inhibitor, LY294002, and all other reagents were purchased from Sigma-Aldrich (St. Louis, MO, USA), unless indicated differently. Antibodies against E-cadherin (sc-7870), *β*-actin (sc-47778), p-Akt (Ser 473; sc-7985-R), total Akt (sc-1618), p-ERK (Tyr204; sc-7383), total ERK (sc-94), p-AMPK (Thr172; sc-33524), and total AMPK (sc-25792), p-Bcl-2 (Ser70; sc-21864-R), p62/SQSTM1 (sc-25575), and beclin-1 (BECN1; sc 11427) were purchased from Santa Cruz Biotechnology Inc. (Santa Cruz, CA., USA); most antibodies against autophagic proteins, Atg5 (NB110-53818), Atg3 (R-159-100), and LC3B (NB100-2220) were purchased from Novus Biologicals (Novus Biologicals, LLC, Littleton, CO, USA); anti-cleaved caspase-3 (cat. number 9661) and p-mTOR (Ser2448; cat. number 5536) were provided by Cell Signaling Technology Inc. (Danvers, MA, USA); anti-Ki-67-FITC conjugated antibody (cat. number 334711) and Alexa Fluor488 secondary antibody (cat. number A21441) were purchased from Life Technologies, Invitrogen (Carlsbad, CA, USA); secondary antibodies IRDye 680 or IRDye 800 CW used for proteins detection by Odyssey Infrared Imaging System (LI-COR Biosciences) were purchased from LI-COR Biosciences (Lincoln, NE, USA). Plastic cell culture plates and flasks were purchased from Corning Incorporated (Lowell, MA, USA). Sterile conical flasks, Lab-Tek Chamber Slides, and disposable pipettes were supplied by Nunc Inc. (Naperville, IL, USA). Growth factor reduced Matrigel was obtained from BD Biosciences (San Jose, CA, USA); each lot of the GFR-Matrigel contained 9–11 mg/mL protein concentration.

### 2.2. Cell Culture

Bovine BME-UV1 mammary epithelial cell line was purchased from the Cell Bank of The Lombardy and Emilia Romagna Experimental Zootechnic Institute, Italy. During routine culture cells were grown in monolayer, on plastic culture flasks, and in growth medium comprising DME/F-12, RPMI-1640, and NCTC 135 in proportions of 5 : 3 : 2 by vol. and enriched with *α*-lactose (0.1%), glutathione (1.2 mM), bovine insulin (1.0 *μ*g/mL), bovine holo-transferrin (5.0 *μ*g/mL), hydrocortisone (1.0 *μ*g/mL), L-ascorbic acid (10 *μ*g/mL), 10% (v/v) heat-inactivated FBS, penicillin-streptomycin (50 IU/mL), fungizone (2.5 *μ*g/mL), and gentamycin (50 *μ*g/mL).

In order to begin 3D culture, plates (100 mm diameter) (Corning Inc., NY, USA) or 8-well, Lab-Tek Chamber Slides (Nunc Inc., Naperville, IL, USA) were coated with growth factor reduced Matrigel (BD Biosciences) and 400 *μ*L of Matrigel was spread on the surface of culture plates or 25 *μ*L (per well) on chamber slides. The plates and chamber slides were left at 37°C for 30 min for Matrigel to solidify. Confluent BME-UV1 cells grown in monolayer were trypsinized and resuspended in growth medium with addition of 2% Matrigel. The cells were plated at a concentration of 25000 cells/mL on culture plates or 5000 cell/mL on chamber slides. After 24 h the medium was replaced with a differentiation medium, containing DME/F-12, RPMI-1640 and NCTC 135 in proportions of 5 : 3 : 2 by vol., enriched with *α*-lactose (0.1%), glutathione (1.2 mM), bovine insulin (1.0 *μ*g/mL), bovine holo-transferrin (5.0 *μ*g/mL), hydrocortisone (1.0 *μ*g/mL), L-ascorbic acid (10 *μ*g/mL), 2% (v/v) heat-inactivated FBS, penicillin-streptomycin (50 IU/mL), fungizone (2.5 *μ*g/mL), gentamycin (50 *μ*g/mL), and 2% GFR-Matrigel. Additionally in experimental conditions, differentiation medium was supplemented with E2 (1 nM), P4 (5 ng/mL), or combination of E2 and P4. Medium was replaced every second day. Growth of the acinar structures formed by BME-UV1 cells was monitored daily using Olympus IX70 inverted phase contrast microscope (Olympus Optical Co., Hamburg, Germany).

### 2.3. Confocal Microscopy

BME-UV1 cells cultured on chamber slides coated with Matrigel were fixed in 3.7% paraformaldehyde (20 min) after 3, 6, 9, or 14 d of culture. Next cells were permeabilized with 0.5% Triton X100 in PBS (10 min), washed three times with PBS, and incubated overnight with primary antibody against E-cadherin, cleaved caspase-3, beclin-1, or p62 diluted 1 : 100 with PBS. After primary incubation the cells were washed three times with PBS and incubated with 1 : 500 Alexa Fluor488 secondary antibodies for 1 h at room temperature. After incubation with appropriate antibodies cells were incubated with 7-aminoactinomycin D (7-AAD), 5 *μ*g/mL for 30 min to counterstain DNA. Finally coverslips were mounted on microscope slides using SlowFade Gold reagent (Life Technologies, Invitrogen). Cells were visualized using the confocal laser scanning microscope FV-500 system (Olympus Optical Co, Hamburg, Germany). The combination of excitation/emission were Argon 488 nm laser with 505–525 nm filter for Alexa Fluor 488 and HeNe 543 nm laser with 610 nm filter for 7AAD nucleus staining. Intensity of immunofluorescence staining of cleaved caspase-3 and p62 was also analyzed quantitatively using MicroImage analysis software (Olympus). Integrated optical density (IOD), which measures optical density of individual pixels in a chosen area, was used in the analysis. IOD of green fluorescence of cleaved caspase-3 or p62 was measured in the total area of each single mammosphere. In parallel IOD of red fluorescence of nuclei was measured in the same area. The data are presented as ratios of green fluorescence IOD to red fluorescence IOD for each analyzed mammosphere. Data were collected for each experimental replicate and underwent statistical analysis described in chapter 2.6. For each time point the presented images are representative of at least three independent experiments.

### 2.4. Real-Time PCR

BME-UV1 cells were grown on GFR-Matrigel-coated culture plates for 3, 6, 9, or 14 d in differentiation medium. Next, cells were pelleted by centrifugation, disrupted in 600 *μ*L of RLT Buffer from the Qiagen RNeasy Mini Kit (cat. number 74104), and stored in −80°C until use. Total RNA was extracted from cells with the RNeasy Mini Kit according to the protocol supplied by the producer (Qiagen, Inc., Mississauga, Canada). RNA concentration and purity were determined spectrophotometrically, and quality was confirmed using microcapillary electrophoresis (Bioanalizer 2100, Agilent Technologies, Mississauga, Canada). During reverse transcription, 2 *μ*g of isolated total RNA was converted to cDNA with High Capacity cDNA Reverse Transcription Kit (Applied Biosystems, California, United States), and the reaction was carried out in a Mastercycler pro (Eppendorf, Germany). Real-time PCR was performed in triplicate using SYBR Select Master Mix (Applied Biosystems). Each 10 *μ*L reaction contained a final concentration of 0.5 *μ*M each of forward and reverse primers, 1x master mix, and 1 *μ*L cDNA (100 ng). Reaction was performed in Mx3005P QPCR machine (Stratagene, La Jolla, CA, USA). Cycling condition started with two initial phases at 50°C for 2 min and 95°C for 2 min, which were followed by 40 cycles: 95°C for 15 sec; 58°C for 15 sec; 72°C for 1 min each, respectively. Standard curves were run for each transcript to ensure exponential amplification, and “no RT” controls were run to exclude nonspecific amplification.* GAPDH* was used as a reference gene. Primers sequences are listed in Table 1. Comparative CT method [12] was used to calculate the fold change in gene expression normalized to reference* GAPDH* gene.

### 2.5. Western Blot Analysis

Cells were grown on GFR-Matrigel-coated culture plates for 3, 6, 9, or 14 d. Next cells were pelleted by centrifugation at 14000 rpm at 4°C for 5 min and frozen at −80°C until further analyses. Protein extracts from cultured cells were isolated by lysing the collected cell pellets with RIPA buffer (50 mM Tris, pH 7.5, 150 mM NaCl, 1 mM EDTA, 1% NP-40, 0.25% Na-deoxycholate, and 1 mM PMSF) supplemented with protease inhibitor cocktail and phosphatase inhibitor cocktail (Sigma-Aldrich) for 30 min at 4°C. Lysates were cleared for 20 min at 14000 rpm, and supernatants were collected. Protein concentration in the lysates was determined using Bio-Rad protein assay dye reagent according to the producer's instructions (Bio-Rad Laboratories Inc., Hercules, CA, USA). Proteins (50 *μ*g) were resolved by SDS-PAGE and transferred onto PVDF membrane (Sigma-Aldrich). For immunostaining membranes were blocked with 5% nonfat dry milk in TBS (20 mM Tris-HCL, 500 mM NaCl) containing 0.5% Tween20. The membranes were incubated with chosen primary antibodies at a dilution range between 1 : 200 and 1 : 1000, depending on the antibody. Next the membranes were washed three times for 15 min and incubated with appropriate secondary antibodies conjugated with IR fluorophores: IRDye 680 or IRDye 800 CW (at 1 : 5000 dilution). Odyssey Infrared Imaging System (LI-COR Biosciences) was used to analyze the protein expression. Scan resolution of the instrument was set at 169 *μ*m and the intensity at 4. Quantification of the integrated optical density (IOD) was performed with the analysis software provided with the Odyssey scanner (LI-COR Biosciences). Immunoblot analysis was performed on samples from three independent experiments. For the purpose of publication the color immunoblot images were converted into black and white images in the Odyssey software.

### 2.6. Statistical Evaluation

Statistical analysis was performed using GraphPad Prism version 5.00 software (GraphPad Software, Inc., La Jolla, CA, USA). In the case of analyzing the IOD results obtained for immunofluorescence staining of cleaved caspase-3 and p62 statistical significance of the mean IOD ratios was calculated using the one-way analysis of variance (ANOVA) and Tukey's multiple comparison posttest. The comparison was made between the treatments (control and different experimental conditions: E2, P4, and E2 + P4) at a specific time point (days 6, 9, and 12). Statistical significance of the mean IOD values for immunoblot bands was calculated using the two-way analysis of variance (two-way ANOVA) and Bonferroni multiple-comparison correction, when the comparison was made between the treatments (control and E2, P4, and E2 + P4) and duration of 3D culture (3, 6, 9, and 14 days). Since in most cases two-way analysis of variance did not show significance of the Western blot results obtained we also performed one-way ANOVA and Tukey's multiple-comparison posttest to analyze the potential differences between experimental conditions (control and E2, P4, and E2 + P4) at specific time point of the experiment (3, 6, 9, or 14 days). *P* value of ≤0.05 was considered statistically significant and *P* ≤ 0.01 or *P* ≤ 0.001 as highly significant.

## 3. Results

### 3.1. Influence of 17*β*-Estradiol and Progesterone on Development of Acini Formed by Bovine MECs Cultured on rBM, Rate of Apoptosis, and Autophagy

**Figure 1:**
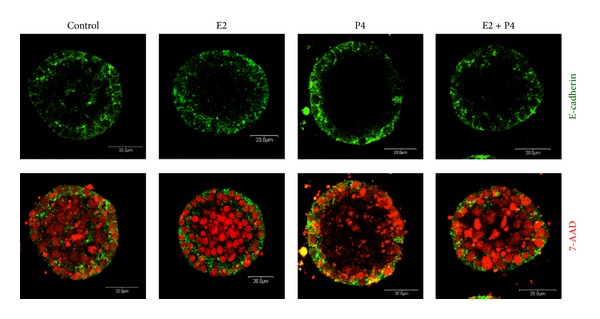
E-cadherin localization in acinar structures formed by BME-UV1 cells cultured on Matrigel for 12 days in differentiation medium (control), enriched with 17*β*-estradiol (E2, 1 nM), progesterone (P4, 5 ng/mL), or both (E2 + P4); E-cadherin (epithelial cell marker) was labeled with antibodies conjugated with Alexa Fluor 488 (green fluorescence) and DNA was counterstained with 7AAD (red fluorescence).

Our previous* in vitro* studies have shown that BME-UV1 bovine mammary epithelial cells cultured on rBM (Matrigel) form alveoli-like structures and their development occurs faster in the presence of sex steroids [8]. Since at the time of gestation both steroids are present in high concentrations, playing an important function during the final stages of mammary gland development, we decided to investigate the individual, as well as simultaneous effects of E2 (1 nM) and P4 (5 ng/mL) in the course of acini formation by bovine MECs cultured in 3D system. Confocal images of spherical structures formed by BME-UV1 cells confirmed our previous observations, showing that proper development of such acini was possible with and without sex steroids. Cells which were directly attached to extracellular matrix (ECM) underwent polarization, and the typical lateral localization of E-cadherin was noted in these cells in all tested conditions (control and with addition of sex steroids) (Figure 1).

**Figure 2:**
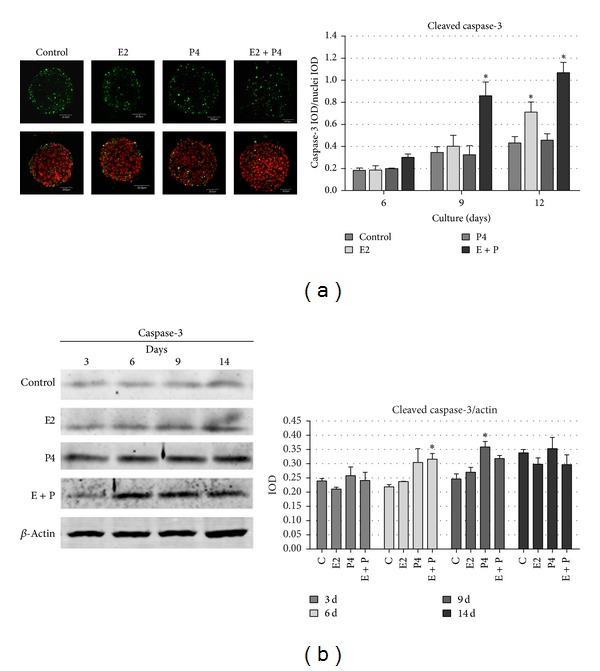
Cleaved caspase-3 expression in BME-UV1 cells cultured on Matrigel in differentiation medium (control), enriched with 17*β*-estradiol (E2, 1 nM), progesterone (P4, 5 ng/mL), or both (E2 + P4) for 3, 6, 9, 12, or 14 days; (a) images present immunofluorescence staining of cleaved caspase-3 (green fluorescence) and DNA counterstained with 7AAD (red fluorescence) and graph beside represents quantitative analysis of the intensity of green fluorescence of cleaved caspase-3 immunostaining, presented as the ratio of integrated optical density (IOD) of caspase-3 to IOD of nuclei in each acinus analysed; (b) Western blot analysis of the levels of cleaved caspase-3 in cell lysates: expression of *β*-actin was used as a loading control; graph beside the image represents the obtained results of densitometric analysis, in which IOD of each band was measured, and the values were normalized to IOD of *β*-actin; the IOD results are presented as means ± SEM from at least three separate experiments; statistically significant differences (*P* < 0.05) in comparison with control conditions were marked with *.

When we analyzed the expression and localization of apoptotic marker, cleaved caspase-3, in developing acini we noted more immunofluorescence staining of active caspase-3 in the center of spherical structures formed in the presence of E2 and P4 than in control conditions. The differences were more evident in the second week of 3D culture, which is the time when the formation of hollow lumens occurs in these structures, as demonstrated in our previous study (Figure 2(a)) [8]. To confirm these observations we used MicroImage analysis software (Olympus) to measure the intensity of green fluorescence (IOD) related to cleaved caspase-3 immunostaining in each analysed acinus from days 6, 9, and 12 of 3D culture and compared it with red fluorescence of nuclei. The results, which are presented as ratios of green fluorescence IOD to red fluorescence IOD, confirmed the time dependent increase in cleaved caspase-3 expression within the developing acini. The highest mean IOD values were obtained in the spherical structures cultured in the presence of E2 and E2 + P4 (Figure 2(a)). However, the addition of both steroids simultaneously seems to create the optimal conditions for acini development, as the activity of caspase-3 in these conditions was significantly increased already on day 9 of 3D culture. Western blot analysis of the levels of cleaved caspase-3 in cell lysates also confirmed our observations, showing that addition of E2 and P4 together resulted in a significant increase in this apoptotic enzyme (Figure 2(b)).

**Figure 3:**
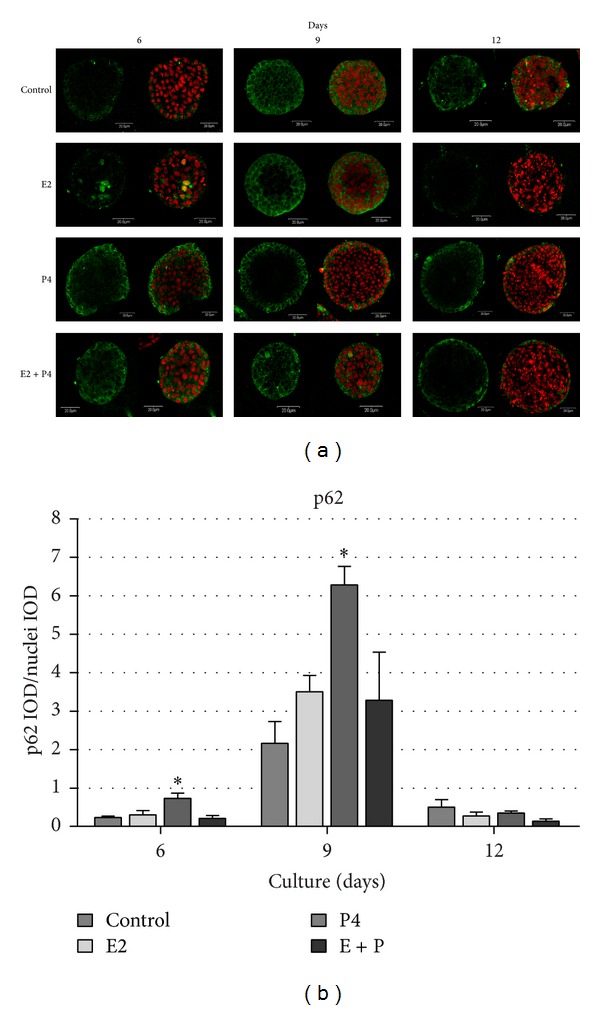
Confocal images of acinar structures formed by BME-UV1 cells cultured on Matrigel for 6, 9, or 12 days in differentiation medium (control), enriched with 17*β*-estradiol (E2, 1 nM), progesterone (P4, 5 ng/mL), or both (E2 + P4); (a) panel of images presents immunofluorescence staining of p62 (green fluorescence): DNA was counterstained with 7AAD (red fluorescence); (b) graph representing quantitative analysis of the intensity of green fluorescence of p62 immunostaining, presented as the ratio of integrated optical density (IOD) of p62 to IOD of nuclei in each acinar structure analysed; *statistically significant difference (*P* < 0.05) in comparison with control conditions.

Our previous findings demonstrated that 17*β*-estradiol and progesterone also enhanced autophagy in the center of developing alveoli formed by BME-UV1 cells, which was manifested by increased punctuated pattern of GFP-LC3-II [8]. To further confirm the role of both sex steroids in regulation of autophagy in bovine MECs in the course of acini development we investigated the expression of p62 in these structures. p62 (also known as sequestome 1-SQSTM1) is a ubiquitin-binding scaffold protein that directly binds both poly- or monoubiquitin via its ubiquitin-associated domain (UBA) and LC3 and links the ubiquitinated cargos to the autophagy machinery for autophagic degradation [13]. The protein itself is also degraded by autophagy. Since p62 accumulates when autophagy is inhibited and decreased levels can be observed when autophagy is induced, p62 may be used as a marker to study autophagic flux [14]. Immunofluorescence staining of p62 revealed high expression of this protein mainly in the outer cells of the acini (Figure 3). In the first week of 3D culture (6 d) the levels of this protein were not high; however, increased expression could be noted in bovine MECs treated with P4 (Figures 3(a) and 3(b)). On day 9 of culture p62 positive staining was increased in all tested conditions, but in the presence of sex steroids the differentiation of two populations of cells with difference in p62 expression was more pronounced. This effect was especially clear after P4 treatment, and densitometric analysis done using MicroImage software confirmed the significant increase of p62 IOD in the presence of progesterone on days 6 and 9. However, these data represent the total fluorescence in the area of the acinar structures, without distinguishing the inner and outer population of cells. On day 14 spherical structures formed in the presence of sex steroids administered separately or together expressed p62 only in the outer cell population, whereas in control cells some green fluorescence could still be noted in the center. These results indicate that E2 and P4 increased the rate of autophagy in the centrally located cells of the acini, manifested by increased degradation of p62 (Figure 3).

### 3.2. Regulation of Atg Proteins Expression in Bovine MECs Cultured on rBM

**Figure 4:**
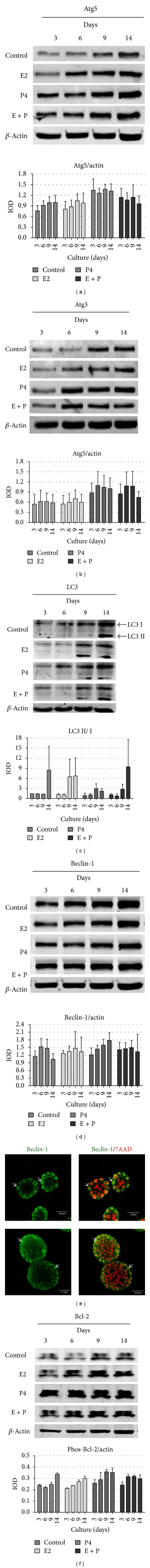
Expression of autophagic proteins (Atg5, Atg3, LC3, and beclin-1) and phosphorylated Bcl-2 (Ser70) in BME-UV1 cells forming acinar structures on Matrigel. Representative images of Western blot analysis of Atg5 (a), Atg3 (b), LC3 (c), beclin-1 (d), and phosphorylated Bcl-2 (Ser70) (f) in bovine MECs grown in 3D culture for 3, 6, 9, and 14 days in differentiation medium (control), enriched with 17*β*-estradiol (E2, 1 nM), progesterone (P4, 5 ng/mL), or both (E + P); expression of *β*-actin was used as a loading control; graphs below the images show the results of densitometric analysis, in which IOD of each band was measured, and the values were normalized to IOD of *β*-actin; the IOD results are presented as means ± SEM from at least three separate experiments. (e) Confocal images of immunofluorescence staining of beclin-1 (green fluorescence) in cells grown in 3D culture for 3 or 9 days in control conditions: DNA was counterstained with 7AAD (red fluorescence) and white arrows indicate the nuclear localization of beclin-1.

In the next step of our study we investigated the potential influence of 17*β*-estradiol and progesterone (administered separately or together) on the level of autophagic proteins involved in autophagosomes formation. Western blot analysis was performed on cell lysates isolated from acini formed by BME-UV1 cells cultured on Matrigel in the presence of E2, P4, or E2 + P4 for 3, 6 9, and 14 days. In control conditions the levels Atg5 and Atg3 increased from day 9 of 3D culture (Figures 4(a) and 4(b)). Our results demonstrated that P4 markedly elevated the amount of Atg5 from the earliest time point (3 d), and this effect was also observed when steroids were added together (Figure 4(a)). In the case of E2, the increase in Atg5 level could also be noted, but from the 6th day of culture; however, densitometric analysis did not confirm these differences (Figure 4(a)). Similar tendencies were observed for Atg3 protein, in which an increase was observed faster in cultures supplemented with steroids (from day 6) than in control conditions (day 9) (Figure 4(b)). Also LC3 II, the active, cleaved form of LC3 protein, was detected from day 9 of 3D culture in the presence of steroid hormones in contrast to control conditions, in which a substantial increase in LC3-II did not appear until day 14 (Figure 4(c)). Interestingly, activation/cleavage of LC3 protein was the most pronounced when bovine MECs were treated with estrogen alone, or both steroids simultaneously, whereas progesterone did not exert such a clear effect. In the case of beclin-1 no distinct difference was observed between cells cultured under control conditions and those treated with E2, P4, and E2 + P4 together (Figure 4(d)). We have noted, however, differences in localization of beclin-1 in cells being in direct contact with rBM and those located in the center of developing acinar structures. Immunofluorescence staining of beclin-1 revealed that the most outer cells had perinuclear localization of beclin-1, whereas inner population of MECs showed cytoplasmic staining (Figure 4(f)). This pattern of staining was similar in all tested conditions. Since beclin-1 needs to be exported from the nucleus to the cytoplasm in order to play its function in autophagy induction our findings further support the hypothesis about the differences between the outer population of cells receiving direct signals from the substratum and the centrally located cells lacking the contact with ECM, thus inducing autophagy and being prone to apoptotic cell death. Additionally, we investigated the level of Bcl-2 protein which forms complexes with beclin-1 inhibiting autophagy. Studies have shown that Bcl-2 is released from these complexes after phosphorylation (at Ser70) [15]; therefore, we analyzed the expression of the phosphorylated form of this protein. The results demonstrated that the levels of phos-Bcl-2 increase in time of 3D culture and sex steroids further elevated the level of this protein. The most pronounced effect was noted when bovine MECs were cultured in the presence of P4, and E2 + P4; however, densitometric analysis did not show statistical significance of these differences (Figure 4(f)).

### 3.3. Regulation of ATGs Expression by 17*β*-Estradiol and Progesterone in Bovine MECs

To investigate whether 17*β*-estradiol and progesterone regulate autophagy during spheroids formation via the classical genomic pathway we analyzed the influence of these steroids on the expression of genes involved in autophagy induction and formation of autophagosomes in MECs. In order to choose candidate genes for real time RT-PCR we performed a preliminary computer analysis of potential binding sites for steroid receptors on promoters of bovine autophagy-related genes using MatInspector software (Genomatix). This analysis revealed that the promoter regions of bovine* ATG5* and* LC3B* genes potentially contain estrogen response element (ERE), whereas* ATG3* shows the presence of androgen response element (ARE). Additionally, other analyzed autophagic genes, such as beclin-1, contained many binding sites for AP1, Sp1, and CREB transcription factors, which are known to interact with steroids receptors, enabling an indirect activation of gene expression by sex steroids [16–19]. On the basis of these preliminary results of* in silico* analysis we chose to investigate the expression of four genes which play a key role in autophagy induction and autophagosomes formation (*BECN1*,* ATG5*,* ATG3*, and* LC3B*) and are potentially regulated by E2 and P4. We investigated the expression levels of these genes in BME-UV1 bovine mammary epithelial cells treated with E2, P4, and both steroid hormones combined in comparison with control conditions on 3, 6, 9, and 14 days of culture (Figure 2). The expression of each* ATG* under control conditions was appointed as 1 at each time point, and the levels of analyzed mRNA were normalized according to the relative* GAPDH* mRNA expression of each sample. A change in expression was considered significant when at least 1.5-fold increase was noted.

**Figure 5:**
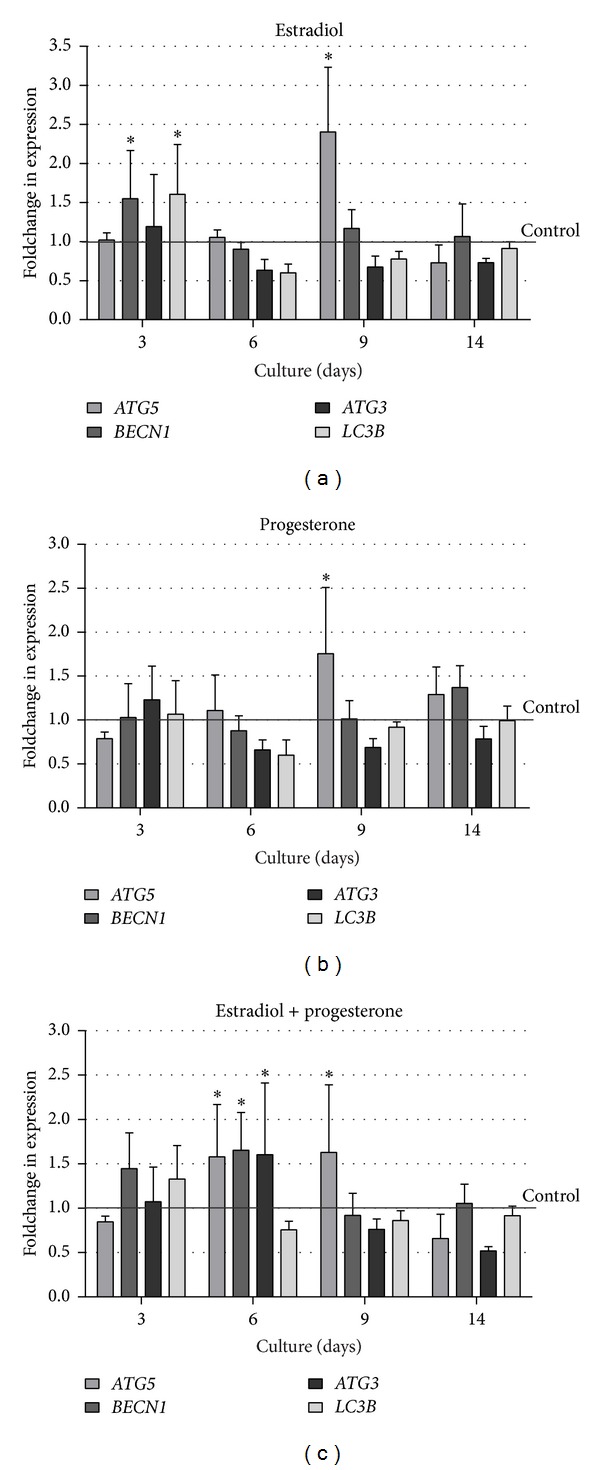
Real-time PCR analysis showing the effects of (a) 17*β*-estradiol (1 nM), (b) progesterone (5 ng/mL), or (c) both steroid hormones administered simultaneously on the expression of chosen autophagy-related genes:* ATG5*,* BECN1*,* ATG3,* and* LC3B* in BME-UV1 cells cultured on Matrigel for 3, 6, 9, and 14 days; the levels of analyzed mRNA were normalized according to the relative* GAPDH* mRNA expression of each sample; the expression of each* ATG* under control conditions was appointed as 1 at each time point; a change in expression was considered significant (*) when at least 1.5-fold increase was obtained.

Real-time PCR results confirmed that* ATG5* was positively regulated by estrogen and progesterone. Over 2-fold increase was noted in cells treated either with E2 or P4, but only on day 9 of 3D culture (Figures 5(a) and 5(b)). It is worth noting, however, that at this time two distinct populations of cells could be distinguished within each acinus: the outer layer of polarized epithelium showing nuclear localization of beclin-1 and sustained expression of p62 protein and the inner cells undergoing stress connected with the gradual loss of contact with ECM. Interestingly, when BME-UV1 cells were treated with both steroids together a significant increase in* ATG5* was observed on the 6th day of culture, suggesting that under these conditions autophagy may be induced earlier (Figure 5(c). Simultaneous presence of E2 and P4 in the culture medium also resulted in significantly increased expression of* ATG3* and* BECN1* on day 6, as well as augmented level of* BECN1* and* LC3B* on day 3 (Figure 5(c)), which points at important interactions between both steroid hormones in regulation of autophagy in bovine MECs. Increased* BECN1* and* LC3B* expression was also noted when BME-UV1 cells were treated only with estrogen (Figure 5(a)); however, these changes were significant only on day 3, whereas progesterone alone did not induce any changes in the levels of transcripts other than* ATG5*.

### 3.4. Analysis of Kinases Activation after Addition of Sex Steroids during Acinar Structures Formation by Bovine MECs

**Figure 6:**
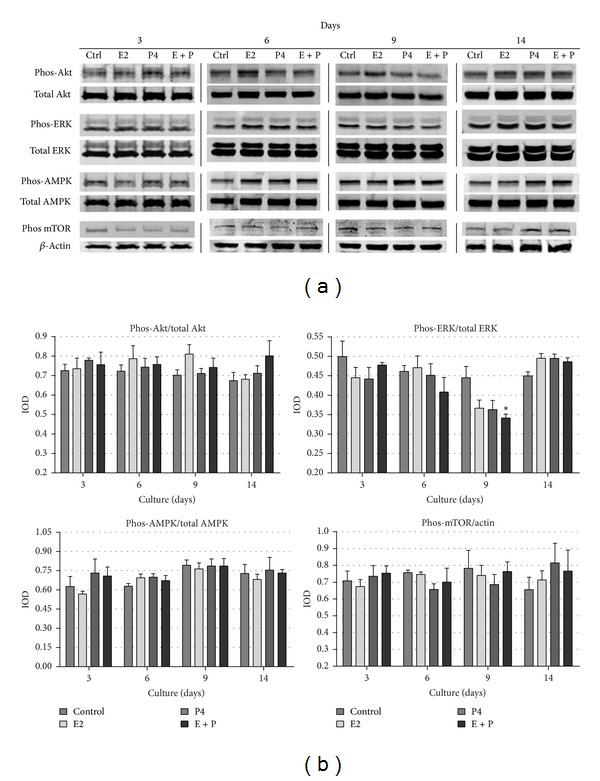
(a) Western blot analysis of the levels of chosen kinases (Akt, ERK, AMPK, and mTOR) involved in autophagy regulation, detected in BME-UV1 cells cultured on Matrigel for 3, 6, 9, and 14 days in differentiation medium (control), enriched with 17*β*-estradiol (E2, 1 nM), progesterone (P4, 5 ng/mL), or both (E + P): expression of *β*-actin was used as a loading control; (b) graphs represent the obtained results of densitometric analysis, in which IOD of each band was measured, and the IOD values for phosphorylated forms of kinases were normalized to the respective IOD of the total forms, with an exception of phos-mTOR, which was normalized to IOD of *β*-actin; results are presented as means ± SEM from at least three separate experiments; *statistically significant difference (*P* < 0.05) in comparison with control conditions.

Estradiol and progesterone are known to mediate their biological actions through specific nuclear receptors: estrogen receptors (ER*α* and ER*β*) and progesterone receptor (PR). Nevertheless, these steroid hormones were also shown to rapidly recruit intracellular signaling pathways, and their action was linked to the transmembrane receptors: G protein-coupled membrane receptor GPR30 [20], membrane progestin receptors (mPR) [21], and progesterone receptor membrane components 1 and 2 (PGRMC) [22]. As a result of activating transmembrane receptors, E2 and P4 mediate their signals through the same cellular pathways as growth factors, mainly MAPK (especially ERK1/2 kinases) and PI3 K/Akt [23]. Downstream signaling pathways mediated through Akt, AMPK, or ERK1/2 kinases may regulate mTORC1 activity, which constitutes the major switch in autophagy induction [24]. Therefore, in our effort to explain regulation of autophagy by sex steroids, we also analyzed the activation pattern of main cellular kinases, which are known to be involved in autophagy induction. The levels of phosphorylated and total Akt, ERK1/2, AMPK, and mTOR kinases were determined by Western blot analysis on days 3, 6, 9, and 14 of 3D culture (Figure 6). In general, no significant changes in the activity of analyzed kinases were observed between the experimental conditions, especially when all performed replicates were analysed densitometrically (Figure 6). Only ERK1/2 activity was significantly reduced on day 9 of 3D culture in cells simultaneously treated with E2 and P4, when compared to control conditions. Steroids seemed to slightly attenuate ERK1/2 phosphorylation (Tyr202) between days 3 and 9 of acini formation by bovine MECs. Interestingly, on day 14 a higher activity of this kinase was noted in all tested conditions, but steroids added separately or together further increased ERK1/2 phosphorylation. Some tendencies could also be noted in the case of Akt kinase activation. At the early time point of 3D culture (day 3) both steroids stimulated phosphorylation of Akt (Ser473), whereas on days 6 and 9 17*β*-estradiol administered alone caused the most pronounced increase in the level of activated Akt in comparison with control, as well as other experimental conditions. This effect seemed to be suppressed by P4 because, in cells treated with both hormones simultaneously, the cellular level of phosphorylated Akt was lower than that observed under control conditions. In the case of AMPK phosphorylation (Thr172) increased levels were noted in the presence of sex steroids on days 3, 6, and 9, when compared with control conditions. The most pronounced effect (although not statistically significant) was observed on day 3 of 3D culture, when BME-UV1 cells were treated with P4 or E2 + P4. Between days 6 and 9 the activity of AMPK further increased in all tested conditions, with slightly higher level in the presence of steroid hormones, and this level was sustained also on day 14 of culture. Finally, E2 and P4 were shown to cause a small decrease in the activity of mTOR kinase (phosphorylation at Ser2448) (on days 3 and 9); however, it was statistically insignificant. It was surprising to find elevated activity of tested kinases on day 14 of 3D culture. This effect might have resulted from the fact that Western blots were performed on lysates from whole spherical structures, which, as mentioned earlier, are composed of two distinct populations of cells: outer population, which remains in favorable conditions thanks to direct interactions with ECM, and inner population, undergoing gradual apoptotic death. On 14th day of culture the number of centrally located cells was largely reduced due to ongoing process of lumen clearance; thus the high activity of kinases might have originated mostly from the remaining living cells forming acini.

## 4. Discussion

In the present study we examined the influence of 17*β*-estradiol (E2) and progesterone (P4) on the expression of autophagic genes and pathways regulating autophagy induction in bovine BME-UV1 mammary epithelial cells cultured on reconstituted basement membrane. The 3D culture model enables recreation of the conditions in which MECs form acinar structures morphologically similar to alveoli that develop during gestation forming the basic secretory units of functionally active mammary gland. The important role of sex steroids in mammary gland development is well documented. Ovariectomy of prepubertal dairy heifers curtails further mammary development, where the earlier in life the procedure is performed, the more drastic is the result [25]. Similarly, mice lacking estrogen receptor (ER) are unable to obtain full glandular development through adulthood, whereas knockout of progesterone receptor (PR) in rodents results in inability to undergo normal lobuloalveolar development [26, 27]. Up to date studies have shown that steroid hormones not only control proliferation of the mammary epithelium, but also are involved in regulation of many different processes, including development, differentiation, and death of mammary epithelium.

Our research demonstrated for the first time that E2 and P4 can regulate autophagy in bovine MECs [7, 8]. It was manifested with increased levels of LC3-II in Western blot analysis, as well as punctuated pattern of GFP-LC3II observed under confocal microscope. When BME-UV1 cells were cultured on rBM the lipidated form of LC3 (LC3-II) was shown to localize predominantly in the center of developing acini. Both E2 and P4 were shown to enhance the rate of spherical structures development by increasing the levels of LC3-II and cleaved caspase-3 [8]. In the present study we further confirmed these observations, showing that in the center of developing acini there is a marked decrease in the levels of p62, whereas cells in direct contact with rBM show sustained expression of this protein (Figure 3). We used p62 as the marker of autophagy, as this scaffold protein is involved in delivering cargo to autophagosomes binds with LC3 and becomes degraded in the course of active autophagy [14]. In parallel we observed central localization of cells expressing cleaved caspase-3 on day 12 of 3D culture (Figure 2). This supports the hypothesis on two distinct populations of cells, whose fate is determined by their localization within the developing mammary acinar structures. Polarized cells are found only in the outer layer because they are able to receive signals from ECM via integrin receptors found on their basal membrane [28, 29], whereas nonpolarized cells comprising the central inner region lack ECM contact and subsequently undergo apoptotic cell death leading to lumen formation [30, 31]. The inner cells also activate autophagy, which has been suggested to be induced due to loss of interactions with ECM [32]. In our study the differences between the two cell populations could also be noted on the basis of beclin-1 localization. The outer layer of bovine MECs expressed beclin-1 predominantly in the perinuclear region, whereas centrally located cells showed cytoplasmic localization of this autophagic protein (Figure 4(e)). Beclin-1 needs to be shuttled from the nucleus to the cytoplasm in order to form the PtdIns3K complex, which initiates nucleation and assembly of the isolating membrane of autophagosomes [13, 33]. Thus, our results demonstrate that only inner cells induce autophagy during acini development.

When BME-UV1 cells cultured on rBM were treated with sex steroids the rate of development of acinar structures was accelerated. We observed increased level of active caspase-3, especially in the presence of progesterone or both steroids together. Also, p62 seemed to be the most actively degraded in the centrally located cells of the acini formed by bovine MECs treated with P4 or E2 + P4, since in these conditions no staining was detected in the inner cells on days 9 and 12 of 3D culture. This supports the general concept of the role of progesterone in alveologenesis. At the same time our findings point at possible important synergistic effects of E2 and P4 in the course of alveoli formation. The synergistic interactions between E2 and P4 signaling in regulation of mammary gland development have been indicated by several studies. Estrogen treatment alone or in combination with progesterone in the intact prepubertal heifers stimulated the proliferation of mammary epithelial and endothelial cells, whereas progesterone treatment alone had no effect on prepubertal mammary cell proliferation [34]. Furthermore, in nonpregnant, nonlactating cows, a combined estrogen and progesterone treatment stimulated MECs differentiation and lobuloalveolar development [35]. Both sex steroids were also shown to stimulate synthesis of glycosaminoglycans (GAGs), which are important ECM components regulating cell proliferation, migration, adhesion, differentiation, and cell-cell communication. A significant increase in total GAGs in the mammary gland of ovary-intact rats administered estradiol singly or in combination with progesterone indicated the stimulatory role of E2 and its synergism with P4 in augmenting total GAGs [36]. In the present study we have shown that simultaneous administration of E2 and P4 during 3D* in vitro* culture of bovine BME-UV1 mammary epithelial cells potentiates the rate of acini development by stimulation of apoptosis and autophagy inside the spherical structures. Since at the time of pregnancy both hormones are present in high concentrations in the mammary gland, it is possible that this coexistence is important for the proper rate of lobuloalveologenesis.

Enhanced autophagy induction in BME-UV1 cells treated with E2 and P4 is related to the ability of these hormones to regulate the expression of autophagy-related genes and their products. It is commonly known that both steroids exert their effect via their nuclear hormone receptors (ER and PR), which function as ligand-bound transcription factors regulating the expression of specific downstream targets [35]. Lu and coworkers [37] performed a microarray study to investigate the transcriptional changes within the mammary gland after administration of E2 or P4, individually and combined. Their results demonstrated that 60% of the differentially expressed genes required combined treatment with E2 and P4, confirming our hypothesis on the synergistic interactions between these sex steroids in regulation of autophagic genes during formation of acini by mammary epithelial cells [37]. However, so far there has been no data showing the possible genomic effect of estrogen and progesterone on the expression of* ATGs*. Our study seems to be the first indicating that both steroids singly as well as combined regulate the expression of* ATG5*, which was significantly increased in all tested conditions on day 9 of 3D culture (Figure 5). Other autophagic genes and their products were also upregulated by sex steroids; however, the effect varied depending on the conditions applied. 17*β*-Estradiol alone elevated the expression of* ATG5*,* BECN1, *and* LC3B* genes, but this effect was observed only at the early time of culture (day 3) and did not fully correspond with the results of Western blot analysis, which did not detect differences in the level of beclin-1 between the tested condition. However, E2 stimulated the formation of LC3-II protein which was increased between days 9 and 14 of the 3D culture of bovine MECs. These results are partially in agreement with the studies performed on other cell types. Yang et al. [38] demonstrated that E2 caused a significant increase in LC3, beclin-1, and ULK1 kinase expression in MC3T3-E1 osteoblastic cells. The promotion of autophagy in osteoblasts by E2 was blocked by ER antagonist, and upregulation of LC3 expression was suppressed by U0126 (ERK kinase inhibitor). The upregulation of* ATG5* and* LC3B* genes by estrogen confirmed the results of our* in silico* analysis, showing that the promoter regions of these genes in the cattle potentially contain estrogen response element (ERE). It was surprising to find, however, that the combination of E2 and P4 abrogated the genomic effect of E2 observed on day 3 but stimulated the expression of* ATG5, ATG3,* and* BECN1* on day 6, maintaining the high expression of* ATG5* also on day 9 of BME-UV1 cells culture. Administration of both steroids together resulted also in increased levels of Atg5 and Atg3 proteins, further supporting the important role of synergistic actions of E2 and P4 in autophagy regulation. It is possible that when E2 and P4 act together on bovine MECs, estrogen predominantly exerts its genomic function, whereas at the same time progesterone regulates the signaling pathways controlling autophagic machinery. Although little is known on the mechanisms of P4 nongenomic effect, some authors reported the rapid actions of this steroid on different cell types. Recent* in vitro* study on neurosteroids, steroid hormones synthesized in central and peripheral nervous system, revealed that progesterone as well as pregnenolone activated autophagy in astrocytes within a few hours after addition [39]. Progesterone activated LC3 protein, and this effect was inhibited by siRNA-mediated knockdown of beclin-1. The neurosteroids regulation of autophagy was proposed to be largely mTOR-independent, and the coexistent activation of mTOR and Akt by P4 probably constitutes a cytoprotective mechanism during autophagy induction in neural cells [39]. Our results also demonstrated increased phosphorylation of Bcl-2 (at Ser70) in the presence of progesterone (Figure 4(f)). Bcl-2 is an antiapoptotic protein, which also functions as autophagy inhibitor [40]. Under nutrient-rich conditions beclin-1 strongly interacts with Bcl-2 inhibiting formation of a multiprotein complex comprising PtdIns3K/Vps34 kinase and beclin-1 (as well as other proteins). The PtdIns3K complex is necessary to initiate autophagosome formation in the cells. In contrast, in starvation, when autophagic rates are high, the interaction between beclin-1 and Bcl-2 is weak. In these conditions Bcl-2 undergoes phosphorylation releasing beclin-1 [15, 41]. During formation of acini by bovine MECs the inner cells deprived of the contact with ECM undergo stress connected not only with the lack of integrin-mediated signaling, but also with undernourishment. In control conditions the levels of phosphorylated Bcl-2 gradually increased on the later days of 3D culture (d 9, 14). In the presence of P4 and E2 + P4 Bcl-2 phosphorylation was augmented already from day 3, suggesting earlier induction of autophagy.

Several other signaling pathways are orchestrated to regulate the dynamic process of autophagy. As a key cellular response activated to compensate extra- or intracellular stress, such as nutrient deprivation, hypoxia, and reduced levels of growth factors, autophagy is regulated by diverse signaling pathways, that induce or inhibit the activity of mTOR kinase [24]. Under normal conditions mTOR remains associated with other proteins in a complex known as mTORC1, leading to increased translation and synthesis of cell cycle regulating and ribosomal proteins [42]. Upon mTOR inhibition by stress, such as starvation, ULK1 and ULK2 (mammalian homologs of Atg1) become activated and phosphorylate Atg13 and FIP200, which are essential for autophagy induction [43]. The activity of mTORC1 complex is controlled by signals from two major sources, the phosphoinositide 3-kinase (PI3K) pathway, an important signaling pathway downstream of receptor tyrosine kinases, and the LKB1/AMPK pathway [44, 45]. PI3K/Akt pathway is induced by growth factors and hormones stimulation, leading to mTOR activation, whereas AMPK (AMP kinase) is a master regulator of cellular energy metabolism, sensing the ATP to AMP ratio and becoming activated when the ATP/AMP ratio is decreased. This in turn leads to inactivation of mTOR and induction of autophagy. The aforementioned pathways play also an important role in development of the mammary gland. PI3K/Akt signaling pathway is associated with induction of mammary epithelial cells' proliferation. Studies with the use of 3D culture system revealed that in the process of acini formation by MECs cultured on rBM lumen formation was associated with PI3K/Akt inhibition in the centrally located cells lacking contact with ECM components, which led to apoptotic cell death and lumen clearance [10]. As previously mentioned, these cells were also shown to induce autophagy [8, 11]. Furthermore, expressing a constitutively active variant of Akt or PI3K in MECs resulted in formation of large, misshapen spherical structures, which failed to elicit lumen [46, 47]. A similar effect was noted in bovine MECs cultured on Matrigel in the presence of IGF-I, which is a known mitogen, acting through PI3K/Akt pathway [8, 48]. Further studies revealed that estrogen can regulate the proper development of such IGF-I-stimulated spheroids, decreasing the activity of Akt kinase in the later stages of acini development, which facilitated lumen clearance [48]. Present research demonstrated that combination of E2 and P4 diminished the activity of Akt in the second week of 3D culture of bovine MECs, which was coincident with the induction of autophagy and initiation of lumen clearance in the acini (Figure 4). An opposite effect was noted on the 3rd day of 3D culture, when all cells showed proliferative activity and exhibited cell-cell contact via adherence junctions (results presented in previous report [49]). In this case 17*β*-estradiol alone or together with progesterone increased phosphorylation of Akt, which may be connected with known mitogenic actions of both steroids in the mammary epithelium.

So far, little is known about the involvement of AMPK-mediated pathway in the course of mammary alveoli formation. We showed that the activity of AMPK increased with the time of 3D culture (between days 3 and 9 in control conditions), indicating that the centrally located cells of developing spheroids undergo stress related to decreased energy levels due to diminished contact with nutrients and oxygen which are not able to easily penetrate inside the structures (Figure 6). Addition of P4 or E2 + P4 caused further increase in AMPK phosphorylation at these time points. Increased AMPK activation, corresponded with the slightly decreased levels of phosphorylated/activated mTOR, as well as diminished phosphorylation of Akt kinase on day 9 of 3D culture treated with both E2 and P4. It is worth noting that at that time phosphorylated ERK1/2 was also decreased, and this effect was significant in the case of simultaneous administration of both steroids. Although it has been demonstrated that estrogenic compounds are able to rapidly activate PI3K/Akt and MAPK pathways [50] via their nongenomic actions, the observed decrease in phosphorylation of both signaling pathways on the second week of acini formation by BME-UV1 cells may be correlated with the aforementioned changes in vitality of the centrally located cells and ongoing processes of autophagy and apoptosis. Surprisingly, we noted a renewed activation of Akt, ERK1/2 and mTOR on day 14 of culture on rBM. Phosphorylation of these kinases was augmented by sex steroids administered separately or together. This effect coincided with the time of final stage of lumen clearance, thus the protein lysates, in which kinases were detected by Western blot, might have originated mainly from the remaining population of polarized cells forming acinar structures.

## 5. Conclusions

In conclusion, we demonstrated that 17*β*-estradiol and progesterone enhance differentiation of bovine mammary epithelial cells forming alveoli-like structures in contact with rBM. Fully developed acini, showing hollow lumens in the center, were formed faster in the presence of both sex steroids simultaneously. This effect could be related to synergistic genomic actions of E2 and P4, which stimulated the expression of autophagy-related genes:* ATG3, ATG5*, and* BECN1, *as well as their protein products at the earlier time points of acini formation. Additionally, our results indicate that enhancement of autophagy in the presence of E2 and P4 can also be partially related to their ability to regulate signaling pathways (PI3K/Akt/mTOR; AMPK/mTOR) involved in autophagy induction.

## Figures and Tables

**Table 1:** Primer sequences of examined *ATGs* and parameters of real-time PCR assay.

Target gene	Nucleotide sequence	Real-time PCR
*ATG5 *	FRD: 5′-TTT GAA TAT GAA GGC ACA CC-3′	SYBR Select Master Mix (catalogue number 4472908; Applied Biosystems) (i) 50°C for 2 min (ii) 95°C for 2 min (iii) 40 cycles of (a) 95°C for 15 sec (b) 58°C for 15 sec (c) 72°C for 1 min
	REV: 5′-TGT AAA CCC ATC CAG AGT TG-3′	
*ATG3 *	FRD: 5′-GGT TGT TCG GCT ATG ATG AG-3′	
	REV: 5′-GGG AGA TGA GGG TGA TTT TC-3′	
*BECN1 *	FRD: 5′-AGT TGA GAA AGG CGA GAC AC-3′	
	REV: 5′-GAT GGA ATA GGA ACC ACC AC-3′	
*MAP1 LC3 B *	FRD: 5′-TTA TCC GAG AGC AGC ATC C-3′	
	REV: 5′-AGG CTT GAT TAG CAT TGA GC-3′	
*GAPDH *	FRD: 5′-CTT CAA CAG CGA CAC TCA-3′	
	REV: 5′-CCA GGG ACC TTA CTC CTT-3′	

Primers were designed using Primer 3 software, on the basis of the bovine sequences from NCBI database and verified using Oligo Calc: Oligonucleotide Properties Calculator (free software available online, provided by Northwestern University) to exclude sequences showing self-complementarity, and BLAST (NCBI, U.S. National Library of Medicine) to exclude possible complementarity to other mRNA templates.
